# The importance of a potential phosphorylation site in enamelin on enamel formation

**DOI:** 10.1038/ijos.2017.41

**Published:** 2017-11-29

**Authors:** Wen-Juan Yan, Pan Ma, Ye Tian, Jing-Ya Wang, Chun-Lin Qin, Jian Q Feng, Xiao-Fang Wang

**Affiliations:** 1 Department of Biomedical Sciences and Center for Craniofacial Research and Diagnosis, Texas A&M University College of Dentistry, Dallas, USA; 2 Department of Endodontics, Nanfan Hospital, Southern Medical University, Guangzhou, China

**Keywords:** ameloblastin, enamel, enamelin, FAM20C, phosphorylation, phosphoserine

## Abstract

Enamelin (ENAM) has three putative phosphoserines (pSers) phosphorylated by a Golgi-associated secretory pathway kinase (FAM20C) based on their distinctive Ser-x-Glu (S-x-E) motifs. *Fam20C*-knockout mice show severe enamel defects similar to those in the *Enam*-knockout mice, implying an important role of the pSers in ENAM. To determine the role of pSer^55^ in ENAM, we characterized ENAM^*Rgsc514*^ mice, in which Ser^55^ cannot be phosphorylated by FAM20C due to an E^57^>G^57^ mutation in the S-x-E motif. The enamel microstructure of 4-week-old mice was examined by scanning electron microscopy. The teeth of 6-day-old mice were characterized by histology and immunohistochemistry. The protein lysates of the first lower molars of 4-day-old mice were analyzed by Western immunoblotting using antibodies against ENAM, ameloblastin and amelogenin. ENAM^*Rgsc514*^ heterozygotes showed a disorganized enamel microstructure, while the homozygotes had no enamel on the dentin surface. The N-terminal fragments of ENAM in the heterozygotes were detained in the ameloblasts and localized in the mineralization front of enamel matrix, while those in the WT mice were secreted out of ameloblasts and distributed evenly in the outer 1/2 of enamel matrix. Surprisingly, the ~15  kDa C-terminal fragments of ameloblastin were not detected in the molar lysates of the homozygotes. These results suggest that the phosphorylation of Ser^55^ may be an essential posttranslational modification of ENAM and is required for the interaction with other enamel matrix molecules such as ameloblastin in mediating the structural organization of enamel matrix and protein-mineral interactions during enamel formation.

## Introduction

Dental enamel is the most highly mineralized hard tissue in the body and is unique in its composition and process of formation. In humans and many other mammals, the enamel formation is artificially classified into presecretion, secretion, and maturation stages. At the secretory stage, the polarized ameloblasts secrete enamel matrix proteins (EMPs) on the dentin surface, including amelogenin (AMEL), ameloblastin (AMBN), and enamelin (ENAM), so on. The EMPs are then proteolytically processed by metalloproteinase-20 (MMP20) at the secretory stage and eventually degraded completely by kallikrein-related peptidase 4(KLK4) at maturation stages.^1^

Evolutionary analyses have classified AMEL, AMBN, and ENAM into a family named ‘secretory calcium-binding phosphoproteins’ (SCPP), which have one or more Golgi casein kinase phosphorylation sites recognized by their distinctive Ser-x-Glu/phospho-Ser (S-x-E/pS) motifs.^2^ Family with sequence similarity 20-member C (FAM20C) is a newly discovered Golgi-associated secretory pathway kinase, and is believed to be the genuine casein kinase phosphorylating the SCPP proteins.^3, 4, 5^ *Fam20C*-knockout mice exhibited hypophosphatemic rickets^6^ and severe enamel defects that are very similar to those in the *Enam*-and *Ambn*-knockout mice,^6, 7, 8^ suggesting that phosphoserines (pSers) in EMPs may have important functional roles.

The consensus sequences of S-x-E motifs in ENAM are highly conserved in vertebrate animals.^9–10^ Three putative pSer sites have been identified in the ENAM of pigs (Ser^53^, Ser^191^, and Ser^216^), mice (Ser^55^, Ser^196^, and Ser^219^) and humans (Ser^54^, Ser^191^, and Ser^216^). In a previous study, Masuya *et al* reported two lines of chemically induced *Enam*-mutant mice, ENAM^*Rgsc395*^ (M100395) and ENAM^*Rgsc514*^ (M100514), in which the first S-x-E motifs mutate into I^55^-x-E^57^ and S^55^-x-G^57^, respectively.^11^ The ENAM^*Rgsc395*^ mutation eliminates pSer^55^ from the S-x-E motif, while the ENAM^*Rgsc514*^ mutation eliminates the potential phosphorylation by changing the motif into an unrecognizable one for FAM20C kinase. The breakage of enamel surfaces in ENAM^*Rgsc395*^ and ENAM^*Rgsc514*^ heterozygotes suggests that the phosphorylation of Ser^55^ might be an important posttranslational modification for ENAM. However, the study by Masuya *et al.* did not characterize the enamel phenotypes in ENAM^*Rgsc514*^ homozygotes, and the biochemical consequence of the potential phosphorylation loss is unclear. In this study, we characterized the enamel defects and the biochemical changes in both ENAM^*Rgsc514*^ heterozygotes and homozygotes.

## Materials and methods

### Animals and genotyping

All animal procedures were approved by the Institutional Animal Care and Use Committee of Texas A&M College of Dentistry (Dallas, TX, USA), and performed in accordance with the National Institutes of Health Guide for the Care and Use of Laboratory Animals.

The ENAM^*Rgsc514*^ (M100514) heterozygous mice were purchased from RIKEN BRC (Ibaraki, Japan). ENAM^*Rgsc514*^ mice have a chemically induced A1745G mutation in the *Enam* gene, resulting in a Glu^57^-to-Gly (E^57^ to G) substitution in the first S-x-E motif of the ENAM protein. A genotyping PCR was performed on tail lysates using primers 5′-TTACGCCTGTGTTGGGTCTT-3′ and 5′-TGGTTTGGCACTAGCTCCTT-3′. The genotypes were determined by restriction digestion on the 715  bp PCR products with EarI (New England Biolabs, MA, USA). The reverse complementary chains of the WT allele were cleaved into 575  bp and a 140  bp fragments, while those of the mutant allele were not cleaved because the mutation had eliminated the EarI restriction site. The WT and heterozygotes showed three fragments (715 bp, 575 bp, and 140 bp), comparing to a single 715 bp fragment in the homozygotes.

### Backscattered scanning electron microscopy

Mandibles collected from the 4-week-old ENAM^*Rgsc514*^ heterozygous, homozygous, and WT mice were fixed in 4% paraformaldehyde at 4 °C overnight and dehydrated through ethanol gradient (70%–100%), followed by embedding in methyl methacrylate. The embedded mandibles were cross-sectioned at the first molar level using a slow-speed diamond saw. The surface of the section was ground smooth using 1200 grade silicon carbide abrasive paper and given a final polish using 0.5 μm diamond paste. The polished enamel surfaces were coated with carbon and examined using field emission scanning electron microscopy (Philips XL30, FEI Company, OR, USA).^12^

### H&E staining and immunohistochemistry

The mandibles collected from 6-day-old mice were fixed in 4% paraformaldehyde at 4 °C for 16 h and then decalcified in 8% ethylene diamine tetraacetic acid (EDTA)/PBS (pH 7.4) at 4 °C for 4 days, followed by paraffin embedding. Five-μm thick serial sections were prepared for H&E and immunohistochemistry (IHC) staining, as we previously described.^8^ The primary antibodies used for IHC staining were: anti-ENAM N-terminus (1:600),^13^ anti-ENAM C-terminus (1:50, SC-33107, Santa Cruz Biotechnology, CA, USA), anti-AMBN N-terminus (1:50, SC-33100, Santa Cruz Biotechnology), anti-AMBN C-terminus (1:600, SC-50534, Santa Cruz Biotechnology), and anti-AMEL (1:600, SC-32892, Santa Cruz Biotechnology). Methyl green was used for the counterstaining of IHC analyses.

### Western immunoblotting

The first lower molars dissected from 4-day-old WT, ENAM^*Rgsc514*^ heterozygous, and ENAM^*Rgsc514*^ homozygous mice were ground into powder in liquid nitrogen and lysed using RIPA buffer (ThermoFisher Scientific, Waltham, MA, USA) containing a proteinase inhibitor cocktail (Roche, Indianapolis, IN, USA). After quantitation by bicinchoninic acid (BCA) protein assay (Thermo Fisher Scientific), the lysates containing equal amounts of total proteins from each group were loaded on sodium dodecyl sulfate polyacrylamide gel (SDS–PAGE) and analyzed by Western immunoblotting using the antibodies anti-ENAMN-terminus (1:1 600),^13^ anti-AMBN C-terminus (1:2 000, SC-50534, Santa Cruz Biotechnology), anti-AMEL (1:2 000, SC-32892, Santa Cruz Biotechnology), and β-ACTIN(1:3 000, SC-47778, Santa Cruz Biotechnology), using the methods described previously.^6, 14, 15, 16^

## Results

### The ENAM^*Rgsc514*^ mice showed severe enamel defects

**Figure 1:**
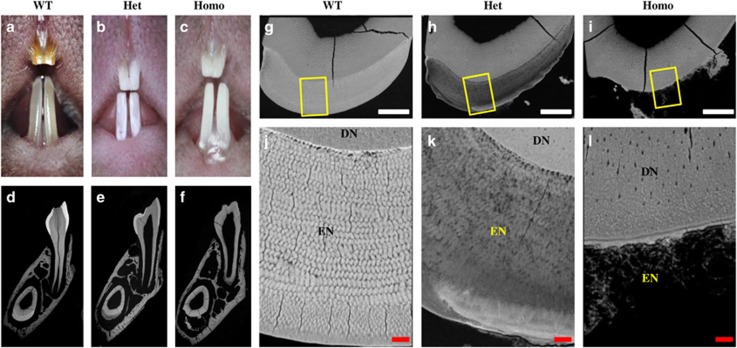
**The gross and microstructural defects of the enamel in ENAM^*Rgsc514*^ mice.** (**a**)–(**c**) At the gross level, the incisors of 7-week-old ENAM^*Rgsc514*^ heterozygotes (Het) and homozygotes (Homo) displayed a chalky white color and rugged surfaces compared to the brownish smooth incisors of the wild type (WT) mice. (**d**)–(**f**) Backscattered SEM images of transversely cut lower jaws from 4-week-old mice. The jaws were cut at the position of the first lower molar. (**g**)–(**i**) Higher magnification of the transversely cut incisors in **d**–**f**. (**j**)–(**l**) Higher magnification of the boxed areas in **g**–**i**. The ENAM^*Rgsc514*^ heterozygotes showed disorganized and hypomineralized enamel rods and interrods compared to the well-organized enamel microstructures in the WT mice; the ENAM^*Rgsc514*^ homozygotes had no enamel on the dentin surface. DN, dentin, EN, enamel. Scale bars: 100 μm in **g**–**i**, 10 μm in **j**–**l**.

The incisors of the ENAM^*Rgsc514*^ heterozygotes and homozygotes had a chalky white color and rugged surface compared with the brown color and smooth surface in the WT mice (Figure 1a–1c). Scanning electron microscopy (SEM) revealed that the well-defined rod and interrod structure of the ENAM^*Rgsc514*^ heterozygotes enamel was less distinct and less mineralized compared with WT mice. Notably, the ENAM^*Rgsc514*^ homozygotes had no enamel on the dentin surface (Figure 1d–1l).

**Figure 2:**
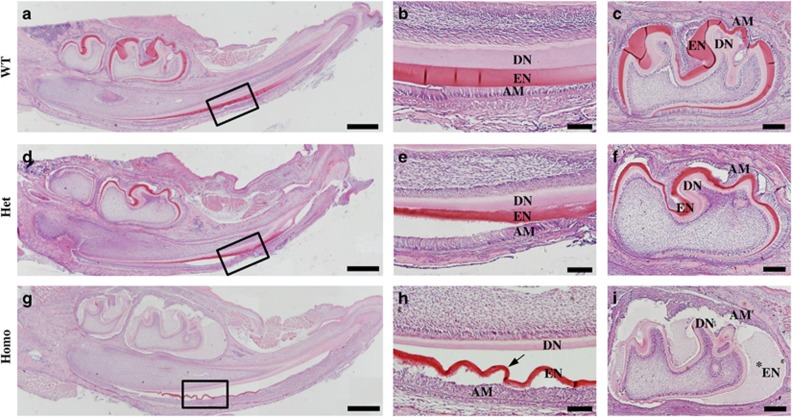
**The histological defects of the enamel in 6-d-old ENAM^*Rgsc514*^ mice.** (**a**)–(**c**) H&E staining of a sagittal section of the lower jaws showed normal histology of dentin (DN), enamel (EN), and ameloblasts (AM) in the lower incisors and the first lower molars in the WT mice. (**d**)–(**f**) In the ENAM^*Rgsc514*^ heterozygotes (Het), both the incisors and molars showed thinner and disturbed enamel matrix compared with that of the WT mice, while their ameloblasts (AM) appeared to be generally normal. (**g**)–(**i**) In ENAM^*Rgsc514*^ homozygotes (Homo), the teeth appeared to be smaller than WT and Het. The ameloblasts lost their polarized shape and detached from the tooth surface. A bubble-like space containing an amorphous substance (asterisk) and/or disorganized enamel matrix (arrow) separated the ameloblasts from the dentin surface. Scale bars: 500 μm in **a**, **d**, and **g**.100 μm in **b**, **e**, and **h**. 200 μm in **c**, **f**, and **i**.

Histological analyses revealed that the ENAM^*Rgsc514*^ heterozygotes formed a thinner and disturbed enamel matrix compared to that in the WT mice (Figure 2a–2f). The ameloblasts of the ENAM^*Rgsc514*^ homozygotes lost their polarized shape and detached from the dentin surface; a bubble-like space between the ameloblasts and the dentin surface was filled with an amorphous substance (Figure 2g–2i). The odontoblasts and dentin of the ENAM^*Rgsc514*^ mice did not show apparent abnormalities.

### The ENAM^*Rgsc514*^ mice showed an altered ENAM distribution pattern

**Figure 3:**
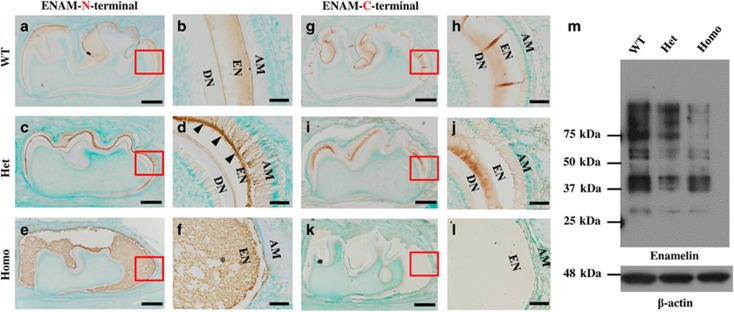
**The distribution and intermediate fragment patterns of ENAM in 6-d-old ENAM^*Rgsc514*^ mice**. (**a**) and (**b**) Immunohistochemcal staining of ENAM in the first lower molars of the 6-d-old WT mice showed that the N-terminal fragments of ENAM were distributed in the outer half of enamel matrix and nearly undetectable in the ameloblasts. (**c**) and (**d**) In ENAM^*Rgsc514*^ heterozygotes (Het), the N-terminal fragments of ENAM were localized at the mineralization front (arrowheads) of enamel matrix (EN) and strongly stained in the cell body of the ameloblasts (AM). (**e**) and (**f**) In the ENAM^*Rgsc514*^ homozygotes (Homo), the N-terminal fragments of ENAM were strongly stained in the scattered amorphous ‘enamel matrix’ (asterisk). (**g**) and (**h**) The C-terminal fragments of ENAM are distributed in the inner half of enamel matrix in the WT mice. (**i**) and (**j**) The C-terminal fragments of ENAM were distributed in the inner ½ of enamel matrix, which is pattern similar to that in the WT mice. (**k**) and (**l**) The C-terminal fragments of ENAM were undetectable in the ‘enamel matrix’ of the ENAM^*Rgsc514*^ homozygotes. (**m**) Western immunoblotting showed that the ENAM^*Rgsc514*^ mice appeared to have less ENAM than the WT mice, while their intermediate fragment patterns are similar. Scale bars: 200 μm in **a**, **c**, **e**, **g**, **i**, and K. 50 μm in **b**, **d**, **f**, **h**, **j**, and **l**.

In 6-day-old ENAM^*Rgsc514*^ heterozygotes, the N-terminal fragments of ENAM were localized at the mineralization front of the enamel matrix and retained in the cell body of the ameloblasts. In contrast, these fragments were evenly distributed in the outer half of the enamel matrix and were nearly undetectable in the ameloblasts in the WT mice (Figure 3a–3f). The distribution pattern of the ENAM C-terminal fragments did not show apparent differences between the WT and ENAM^*Rgsc514*^ heterozygotes. However, these fragments were not detected in the enamel matrix of the ENAM^*Rgsc514*^ homozygotes (Figure 3g–3l). Western immunoblotting revealed that both theENAM^*Rgsc514*^ heterozygotes and homozygotes had less ENAM N-fragments than those in the WT mice, while their cleavage patterns did not show apparent differences among these animals (Figure 3m). We were not able to detect ENAM C-terminal fragments with Western blot using the anti-ENAM-C antibodies.

### The ENAM^*Rgsc514*^ homozygotes showed an altered AMBN cleavage pattern

**Figure 4:**
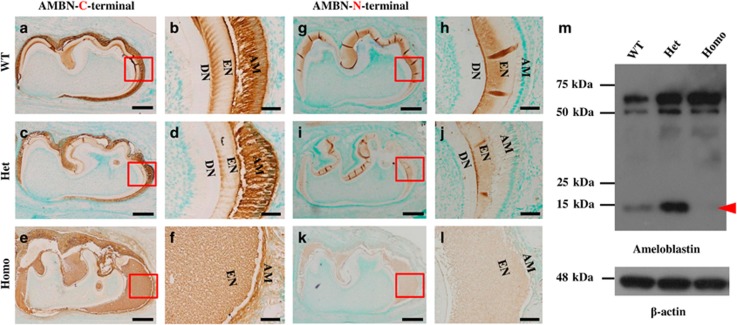
**The distribution and intermediate fragment patterns of AMBN in 6-d-old ENAM^*Rgsc514*^ mice.** (**a**)–(**l**) Immunohistochemical staining of AMBN in the first lower molars of 6-d-old mice showed that the distribution patterns of the C-terminal and N-terminal fragments of AMBN had no apparent differences among the WT and ENAM^*Rgsc514*^ mice. (**m**) Western immunoblotting revealed that a ~15 kDa C-terminal fragment of AMBN (red arrowhead) was absent in the tooth of the ENAM^*Rgsc514*^ homozygotes. Scale bars: 200 μm in **a**, **c**, **e**, **g**, **i**, and **k**. 50 μm in **b**, **d**, **f**, **h**, **j**, and **l**.

The distribution pattern of AMBN N- or C-terminal fragments in the teeth did not show apparent differences between the ENAM^*Rgsc514*^ and WT mice at postnatal 6 days (Figure 4a–4l). However, the cleavage pattern of AMBN displayed an unexpected change in the ENAM^*Rgsc514*^ mice; a~15  kDa AMBN C-terminal fragment was not detected in the ENAM^*Rgsc514*^ homozygotes compared to the WT (Figure 4m). In addition, the ENAM^*Rgsc514*^ mice appeared to have more AMBN expression in the teeth than did the WT mice (Figure 4m).

**Figure 5:**
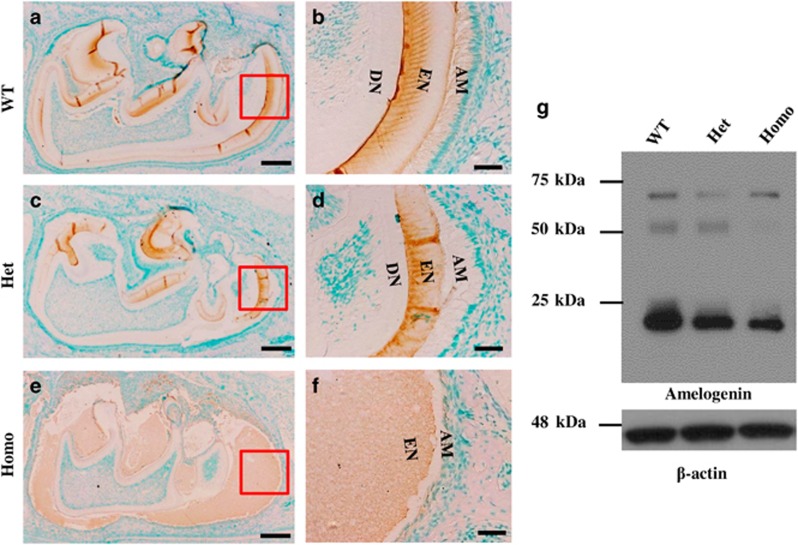
**The distribution and intermediate fragment patterns of AMEL in 6-d-old ENAM^*Rgsc514*^ mice.** (**a**)–(**f**) Immunohistochemical staining of AMEL in the first lower molars of 6-d-old mice showed that the distribution patterns of AMEL had no apparent differences among the WT and ENAM^*Rgsc514*^ mice. (**g**) Western immunoblotting showed similar fragment patterns of AMEL among the WT and ENAM^*Rgsc514*^ mice, although the ENAM^*Rgsc514*^ homozygotes seemingly had less aggregated AMEL at ~50 kDa. In addition, the ENAM^*Rgsc514*^ mice showed fewer amounts of ~20 kDa AMEL fragments compared to the WT mice. Scale bars: 200 μm in **a**, **c**, and **e**. 50 μm in **b**, **d**, and **f**.

The distribution and cleavage patterns of AMEL did not show apparent differences between the ENAM^*Rgsc514*^ and WT mice, while the ENAM^*Rgsc514*^ mice appeared to have less AMEL expression in the teeth compared with the WT mice (Figure 5).

## Discussion

FAM20C has been identified as the Golgi-associated secretory pathway kinase that phosphorylates the serine residues in the S-x-E motifs of ENAM, AMBN, and AMEL.^4, 5^ *Fam20C*-knockout mice showed severe enamel defects similar to those in the *Enam*-knockout mice,^6, 7, 8^ suggesting that the phosphoserines may have an important role in ENAM function. Mammalian ENAMs have three highly conserved pSer residues believed to be phosphorylated by FAM20C.^5, 10^ One of these pSers is located at the N terminus of ENAM, while the other two reside in the 32 kDa “functional” fragments. A previous study has demonstrated that the last pSer in the 32 kDa fragment may have an important role in amelogenesis, as a substitutive mutation of the serine residue (p.S216L) led to hypoplastic AI in humans.^17^ However, it remains unclear whether the AI was associated with the phosphorylation loss or the amino acid change at this residue, as serine and leucine are very different amino acids. A similar question arose when we tried to explain the enamel defects in ENAM^*Rgsc514*^ mice. Theoretically, the E57G substitution prevents FAM20C catalyzed phosphorylation on Ser^55^. However, the residue substitution may be sufficient to cause AI, as Glu and Gly are very different amino acids. The first scenario suggests that the phosphorylation of Ser^55^ is an essential posttranslational modification of ENAM, while the second scenario may suggest that the conserved residues in this area are non-replaceable for normal ENAM functions. Future studies are warranted to discern between these possibilities by substituting S^55^ or E^57^ with amino acids having similar properties, such as A^55^ or D^57^, respectively.

Under normal conditions, ENAM is secreted immediately after being synthesized and thus can barely be detected in the cell body of ameloblasts by IHC staining (Figure 3a and 3b). The full-length ENAM is cleaved into intermediate fragments by MMP20 and transported into specific locations in the enamel matrix: the N-terminal fragments are located in the newly formed enamel matrix, while the C-terminal fragments reside in more mature enamel matrix (Figure 3a–3d).^18–19^ It is important that each step of ENAM secretion, cleavage, and transportation be done correctly in order to form normal enamel. In the ENAM^*Rgsc514*^ mice, the fragmentation of ENAM showed a pattern similar to that in the WT mice (Figure 3m), suggesting that the E57G substitution (or the phosphorylation failure of pSer^55^) may not significantly affect ENAM cleavage. However, the ENAM^*Rgsc514*^ mice showed an abnormal transportation of N-terminal ENAM fragments compared with the WT: the N-fragments were localized at the mineralization front of the newly formed enamel matrix and strongly stained in the cell body of the ameloblasts in the ENAM^*Rgsc514*^ heterozygotes (Figure 3e and 3f) compared with the normal distribution in WT mice (Figure 3a and 3b), suggesting that the conserved residues in this region or the phosphorylation of pSer^55^ is essential for the normal seceration of ENAM N-terminal fragments. We were not able to evaluate the distribution pattern of the N-fragments in the ENAM^*Rgsc514*^ homozygotes, as their ameloblasts were malformed and the enamel matrix was amorphous (Figure 3i and 3j). We did not detect the C-terminal fragments of ENAM in the amorphous enamel matrix of the ENAM^*Rgsc514*^ homozygotes (Figure 3k and 3l). It is unclear if the negative staining suggests an accelerated degradation or an aberrant transportation of the C-fragments. Future studies are warranted to trace ENAM fragments by protein labeling and clarify the role of pSer^55^ by substituting it with more similar/specific residues.

Ameloblastin (AMBN) is the second most abundant protein in the enamel matrix.^20, 21, 22, 23^ MMP20 initially cleaves AMBN at one of the three sites (after Gln^130^, Arg^170^, or Ala^222^), then further cleaves the intermediate fragments at secondary sites near the C-terminus.^22^ The intact AMBN and cleaved fragments have different functions in certain compartments of the developing enamel layers.^24–25^ In general, the N-terminal cleavage forms 13-,15-, and 17-kDa AMBN fragments that accumulate in the sheath space throughout the enamel layer,^22, 26^ while the intact AMBN (62-kDa) and its C-terminal cleavage products are located in superficial enamel and are undetectable below a depth of 50 μm.^27–28^ In this study, the ENAM^*Rgsc514*^ mice did not show apparent differences in the AMBN distribution pattern compared with that in the WT mice (Figure 4a–4l). However, we did not detect the ~15 kDa fragments of the C-terminal AMBN in the molar lysates prepared from the ENAM^*Rgsc514*^ homozygotes by Western immunoblotting compared to those in the WT and ENAM^*Rgsc514*^ heterozygotes (Figure 4m), suggesting that the cleavage of AMBN (which was probably mediated by MMP20) may require the phosphorylation of ENAM. Given that the N-fragments of ENAM (which contain the pSer^55^) showed an overlapped distribution in the newly formed enamel matrix with the C-fragments of AMBN, and that the EMPs coordinate during matrix assembly,^29^ we envisage that pSer^55^ at the ENAM N-terminus may be essential to the structural organization of the enamel matrix and the protein–mineral interactions during enamel formation. Future work is warranted to test this hypothesis by examining the cleavage patterns of AMBN in *Enam*-knockout mice, and the coordination among normal and mutant ENAM, AMBN, and MMP20 during macromolecular assembly, as well as the enzymatic cleavage of AMBN by MMP20 in the presence or absence of phosphorylation in ENAM.

Taken together, we hypothesize that the phosphorylation of Ser^55^ is likely an essential PTM of ENAM, which is probably needed for the interaction with other EMPs, such as AMBN, in mediating the structural organization of the enamel matrix and the protein–mineral interactions during enamel formation.

## References

[bib1] Hu JC, Chun YH, Al Hazzazzi T et al. Enamel formation and amelogenesis imperfecta. Cells Tissues Organs 2007; 186 (1): 78–85. 17627121 10.1159/000102683

[bib2] Kawasaki K, Weiss KM. Mineralized tissue and vertebrate evolution: the secretory calcium-binding phosphoprotein gene cluster. Proc Natl Acad Sci USA 2003; 100 (7): 4060–4065. 12646701 10.1073/pnas.0638023100 PMC153048

[bib3] Ishikawa HO, Xu A, Ogura E et al. The Raine syndrome protein FAM20C is a Golgi kinase that phosphorylates bio-mineralization proteins. PLoS One 2012; 7 (8): e42988. 22900076 10.1371/journal.pone.0042988 PMC3416761

[bib4] Tagliabracci VS, Engel JL, Wen J et al. Secreted kinase phosphorylates extracellular proteins that regulate biomineralization. Science 2012; 336 (6085): 1150–1153. 22582013 10.1126/science.1217817 PMC3754843

[bib5] Cui J, Xiao J, Tagliabracci VS et al. A secretory kinase complex regulates extracellular protein phosphorylation. Elife 2015; 4: e06120. 25789606 10.7554/eLife.06120 PMC4421793

[bib6] Wang X, Wang S, Lu Y et al. FAM20C plays an essential role in the formation of murine teeth. J Biol Chem 2012; 287 (43): 35934–35942. 22936805 10.1074/jbc.M112.386862 PMC3476261

[bib7] Hu JC, Hu Y, Smith CE et al. Enamel defects and ameloblast-specific expression in Enam knock-out/lacz knock-in mice. J Biol Chem 2008; 283 (16): 10858–10871. 18252720 10.1074/jbc.M710565200 PMC2447669

[bib8] Wang SK, Samann AC, Hu JC et al. FAM20C functions intracellularly within both ameloblasts and odontoblasts *in vivo*. J Bone Miner Res. 2013; 28 (12): 2508–2511. 23703840 10.1002/jbmr.1990 PMC3836880

[bib9] Hu JC, Yamakoshi Y, Yamakoshi F et al. Proteomics and genetics of dental enamel. Cells Tissues Organs 2005; 181 (3/4): 219–231. 16612087 10.1159/000091383

[bib10] Al-Hashimi N, Lafont AG, Delgado S et al. The enamelin genes in lizard, crocodile, and frog and the pseudogene in the chicken provide new insights on enamelin evolution in tetrapods. Mol Biol Evol 2010; 27 (9): 2078–2094. 20403965 10.1093/molbev/msq098

[bib11] Masuya H, Shimizu K, Sezutsu H et al. Enamelin (Enam) is essential for amelogenesis: ENU-induced mouse mutants as models for different clinical subtypes of human amelogenesis imperfecta (AI). Hum Mol Genet 2005; 14 (5): 575–583. 15649948 10.1093/hmg/ddi054

[bib12] Wang X, Wang J, Liu Y et al. The specific role of FAM20C in dentinogenesis. J Dent Res 2015; 94 (2): 330–336. 25515778 10.1177/0022034514563334 PMC4300304

[bib13] Brookes SJ, Kingswell NJ, Barron MJ et al. Is the 32-kDa fragment the functional enamelin unit in all species? Eur J Oral Sci 2011; 119 (Suppl1): 345–350. 10.1111/j.1600-0722.2011.00869.x PMC3427898 22243266

[bib14] Wang X, Hao J, Xie Y et al. Expression of FAM20C in the osteogenesis and odontogenesis of mouse. J Histochem Cytochem 2010; 58 (11): 957–967. 20644212 10.1369/jhc.2010.956565 PMC2958138

[bib15] Tian Y, Ma P, Liu C et al. Inactivation of Fam20B in the dental epithelium of mice leads to supernumerary incisors. Eur J Oral Sci 2015; 123 (6): 396–402. 26465965 10.1111/eos.12222 PMC4662618

[bib16] Yang X, Yan W, Tian Y et al. Family with sequence similarity member 20C is the primary but not the only kinase for the small-integrin-binding ligand N-linked glycoproteins in bone. FASEB J 2016; 30 (1): 121–128. 26324849 10.1096/fj.15-273607 PMC4684535

[bib17] Chan HC, Mai L, Oikonomopoulou A et al. Altered enamelin phosphorylation site causes amelogenesis imperfecta. J Dent Res 2010; 89 (7): 695–699. 20439930 10.1177/0022034510365662 PMC2889023

[bib18] Dohi N, Murakami C, Tanabe T et al. Immunocytochemical and immunochemical study of enamelins, using antibodies against porcine 89-kDa enamelin and its N-terminal synthetic peptide, in porcine tooth germs. Cell Tissue Res 1998; 293 (2): 313–325. 9662654 10.1007/s004410051123

[bib19] Hu JC, Hu Y, Lu Y et al. Enamelin is critical for ameloblast integrity and enamel ultrastructure formation. PLoS One 2014; 9 (3): e89303. 24603688 10.1371/journal.pone.0089303 PMC3945975

[bib20] Paine ML, Wang HJ, Luo W et al. A transgenic animal model resembling amelogenesis imperfecta related to ameloblastin overexpression. J Biol Chem 2003; 278 (21): 19447–19452. 12657627 10.1074/jbc.M300445200

[bib21] Fukumoto S, Kiba T, Hall B et al. Ameloblastin is a cell adhesion molecule required for maintaining the differentiation state of ameloblasts. J Cell Biol 2004; 167 (5): 973–983. 15583034 10.1083/jcb.200409077 PMC2172447

[bib22] Chun YH, Yamakoshi Y, Yamakoshi F et al. Cleavage site specificity of MMP-20 for secretory-stage ameloblastin. J Dent Res 2010; 89 (8): 785–790. 20400724 10.1177/0022034510366903 PMC2909333

[bib23] Jacques J, Hotton D, De la Dure-Molla M et al. Tracking endogenous amelogenin and ameloblastin *in vivo*. PLoS One 2014; 9 (6): e99626. 24933156 10.1371/journal.pone.0099626 PMC4059656

[bib24] Bartlett JD, Simmer JP. Proteinases in developing dental enamel. Crit Rev Oral Biol Med 1999; 10 (4): 425–441. 10634581 10.1177/10454411990100040101

[bib25] Simmer JP, Hu JC. Expression, structure, and function of enamel proteinases. Connect Tissue Res 2002; 43 (23): 441–449. 12489196 10.1080/03008200290001159

[bib26] Uchida T, Tanabe T, Fukae M et al. Immunochemical and immunohistochemical studies, using antisera against porcine 25 kDa amelogenin, 89 kDa enamelin and the 13–17 kDa nonamelogenins, on immature enamel of the pig and rat. Histochemistry 1991; 96 (2): 129–138. 1917569 10.1007/BF00315983

[bib27] Murakami C, Dohi N, Fukae M et al. Immunochemical and immunohistochemical study of the 27- and 29-kDa calcium-binding proteins and related proteins in the porcine tooth germ. Histochem Cell Biol 1997; 107 (6): 485–494. 9243282 10.1007/s004180050136

[bib28] Yamakoshi Y, Tanabe T, Oida S et al. Calcium binding of enamel proteins and their derivatives with emphasis on the calcium-binding domain of porcine sheathlin. Arch Oral Biol 2001; 46 (11): 1005–1014. 11543707 10.1016/s0003-9969(01)00070-x

[bib29] Fan D, Du C, Sun Z et al. *In vitro* study on the interaction between the 32 kDa enamelin and amelogenin. J Struct Biol 2009; 166 (1): 88–94. 19263522 10.1016/j.jsb.2009.01.003 PMC4507495

